# Job satisfaction and motivation of health workers in public and private sectors: cross-sectional analysis from two Indian states

**DOI:** 10.1186/1478-4491-8-27

**Published:** 2010-11-25

**Authors:** David H Peters, Subrata Chakraborty, Prasanta Mahapatra, Laura Steinhardt

**Affiliations:** 1 Johns Hopkins University Bloomberg School of Public Health, Baltimore, USA; 2 Jaipuria Institute of Management, Lucknow, India; 3 The Institute of Health Systems, Hyderabad, India

## Abstract

**Background:**

Ensuring health worker job satisfaction and motivation are important if health workers are to be retained and effectively deliver health services in many developing countries, whether they work in the public or private sector. The objectives of the paper are to identify important aspects of health worker satisfaction and motivation in two Indian states working in public and private sectors.

**Methods:**

Cross-sectional surveys of 1916 public and private sector health workers in Andhra Pradesh and Uttar Pradesh, India, were conducted using a standardized instrument to identify health workers' satisfaction with key work factors related to motivation. Ratings were compared with how important health workers consider these factors.

**Results:**

There was high variability in the ratings for areas of satisfaction and motivation across the different practice settings, but there were also commonalities. Four groups of factors were identified, with those relating to job content and work environment viewed as the most important characteristics of the ideal job, and rated higher than a good income. In both states, public sector health workers rated "good employment benefits" as significantly more important than private sector workers, as well as a "superior who recognizes work". There were large differences in whether these factors were considered present on the job, particularly between public and private sector health workers in Uttar Pradesh, where the public sector fared consistently lower (*P *< 0.01). Discordance between what motivational factors health workers considered important and their perceptions of actual presence of these factors were also highest in Uttar Pradesh in the public sector, where all 17 items had greater discordance for public sector workers than for workers in the private sector (*P *< 0.001).

**Conclusion:**

There are common areas of health worker motivation that should be considered by managers and policy makers, particularly the importance of non-financial motivators such as working environment and skill development opportunities. But managers also need to focus on the importance of locally assessing conditions and managing incentives to ensure health workers are motivated in their work.

## Background

Until recently, human resources have been overlooked during the course of health sector reforms [1,2]. The Joint Learning Initiative, World Health Organization, and the Global Health Workforce Alliance have been focusing attention on health workers, particularly on the pervasive problems with staffing shortages, poor job conditions, low remuneration, and extensive migration [3,4]. As the backbone of the health system, health workers usually account for the largest share of public expenditures on health. The presence of high-quality, motivated staff is a key aspect of health system performance, but also one of the most difficult inputs to ensure [5,6]. Health worker job satisfaction, which can be defined as 'the attitude towards one's work and the related emotions, beliefs, and behaviour', results from complex interactions between on-the-job experience, organizational environment, and motivation. Job satisfaction is inextricably linked to motivation, and both involve cognitive, affective, and behavioural processes, with worker motivation commonly understood as the reason why workers behave as they do towards achieving personal and organizational goals. Neither job satisfaction nor motivation is directly observable, but both have been identified as critical to the retention and performance of health workers [7-9]. Some authors contend that the main determinant of health sector performance is health worker motivation, and while resource availability and worker competence are necessary, they are not sufficient [10]. In addition to technical training, health workers must work in environments with incentives in place that reward high-quality performance. To this end, an understanding of employee motivation is necessary to design systems with the right incentives.

In India, low job satisfaction among health workers in the public sector is evident from the highest reported rates of absenteeism of any country [11], while concerns persist about the performance and motivations of a heterogeneous private health sector [12]. Notwithstanding the importance of understanding worker satisfaction in the local context, though there is relatively little empiric information about health worker job satisfaction, motivation, and performance in many developing countries [13,14], and in India in particular.

The purpose of this study is to identify important aspects of health workers' job satisfaction and motivation in different settings in two states in India: Andhra Pradesh (AP) and Uttar Pradesh (UP). The study identifies and assesses differences in the perceived importance and actual presence of job characteristics related to motivation of doctors and nurses in both public and private sectors in the two states. The study was part of a larger research program conducted by Indian institutions to examine options for India's future health systems, with AP and UP representing distinct regions of the country [12]. Identifying the distinct conditions that affect health worker satisfaction and motivation in each sector and state can provide a basis for considering policies and management approaches to improve work conditions that are different in each setting.

Previous analysis on public sector health workers argue that the lack of adequate remuneration is the main health worker grievance in low income countries, and the main reason why public sector health workers are frequently missing or working elsewhere [15]. The Joint Learning Initiative Report also highlights the importance of management culture and working conditions in affecting motivation, and cites small studies in four African and Asian countries that show how motivating factors differ widely, with factors other than remuneration having higher importance [16].

The 2006 World Health Report identified ten major strategies to improve the performance of health workers, including those related to improving job conditions and providing supportive supervision [4]. Paying health workers sufficiently and on-time were also identified as necessary for improving motivation of health workers, particularly to recruit and retain staff, and to prevent absenteeism and collection of informal payments from patients. Efforts to improve health worker motivation have focused on financial incentives, including pay-for-performance [17,18], particularly since wages for health workers tend to be low [4]. Yet well-intentioned efforts to improve financial incentives for health workers can actually undermine morale and lead to negative consequences for health workers [19,20]. Zimbabwe recently made a more concerted effort to address public health sector worker motivation through a series of reforms, including financial reforms, management strengthening, decentralization, and contracting out. However, the mismanaged reform implementation process and the government's poor communication with health workers undermined the potential positive impacts of the reforms [21].

### Theories on health worker motivation

The factors affecting worker satisfaction and motivation has an extensive literature and many theories, some of which has been reviewed by Dolea and Adams [22]. In his seminal work on the *Principles of Scientific Management*, Frederick Taylor advocated providing financial incentives to workers and breaking down work to the one best way to perform tasks to increase their productivity [23], an approach that frequently led to worker resentment and strikes [24]. Content theories were later developed to link worker motivation to the satisfaction of needs. Motivating factors related to job content or other factors related to the job context are seen as contributing to job satisfaction [25-27]. Process theories emphasized subjective expectations or the values of workers as influencing their motivation and work effort [28-30]. Kanfer builds on these theories to stress the importance of employees' willingness and ability to carry out the goals of the organization in which they work [31]. Job characteristics have been identified as critical determinants of health worker motivation and satisfaction [7,8], and have also been described as a core domain in the measurement of health worker motivation, along with organizational commitment and conscientiousness [9]. In this study, we build on these theories to assess workers' subjective assessments of job satisfaction related to the content of the job and its context, adapting an instrument called the Job Descriptive Index (JDI) [32]. We focus on those motivating factors in the work environment that are amenable to change, and contrast workers' assessment of how important these factors are to them with their assessment of how satisfied they are with their current conditions.

### Indian context

India has a rapidly growing economy, yet is still a low income country with high levels of poverty and a complex health system with considerably different conditions prevailing in its different states [33]. Though they are both considered major states in India (AP's population is estimated at 76 million, UP at 166 million, as of 2001), AP is further advanced in the health transition than UP, as shown by lower child mortality rates (86 deaths per 1000 births in children under 5 in AP, versus 123 in UP) and fertility levels (2.5 children per women in AP versus 4.8 in UP) [33]. Compared to other Indian states, both AP and UP provide less than the median level of public spending on health, both are regressive to the poor in their public spending on health, and both have large private sectors providing ambulatory and inpatient hospital care, which continues to attract health workers from the public sector [33]. AP is considered a friendlier environment for private business than UP, and has reaped greater benefits from the rapid expansion of technology, so that work environments are expected to differ. In both states, the public health sector has suffered from inadequate investment and support for operations, adversely affecting work conditions. This has undermined the ability of health workers to deliver high quality care in the public sector, and provides a rationale for informal payments and absenteeism as a means of securing a higher income. Regulation of the private sector is weak throughout India [34], and many private organizations have created work environments that deliver poor quality health care in both AP [35] and UP [36], a situation which is not encouraging to those health workers aspiring to high professional standards. On the other hand, the financial "bottom-line" is a much bigger concern in private sector organizations, and the earning potential is expected to be considerably higher than in the public sector.

## Methods

The studies in each state were conducted by different research organizations as part of larger studies on the health sector. Although each location examined slightly different research topics, the job satisfaction part of the study followed a common protocol. A sampling frame of health providers did not exist in either state, and each organization needed to take a slightly different approach to arrive at random samples of health workers.

In the case of AP, a database of public and private facilities was held by the Institute of Health Systems, which was updated through interviews with practicing local doctors, and officials from medical colleges, professional associations, and other agencies. Three strata of districts were purposely identified to represent different socioeconomic areas of the state, with one district selected at random from each stratum. Three large hospitals (those with over 100 beds), 20 small hospitals, and 25 primary health clinics and/or solo practitioners were randomly selected from the lists of public and private facilities in each district, using a random starting point and fixed selection interval. Doctors and nurses were selected at random from a list of all doctors and nurses usually working at each facility, according to the size of the facility (up to four doctors and two nurses at large hospitals, up to four health workers at small hospitals, and one for solo practitioners). Names of public providers who may have been assigned to a facility but had never or rarely showed up, or who were on official leave, were not included in our sample. In UP, one district was randomly selected from each of three strata of districts representing different socioeconomic areas of the state, as was done in AP. A list of public facilities had been updated by the state's Department of Health and Family Welfare, and a random sample was taken of 70 large hospitals, 70 small hospitals, and 100 primary health centers, with health providers randomly selected from a list of providers in a similar manner to AP. A larger sample was taken because it was part of a baseline evaluation of a quality improvement project in the public sector, which was conducted by a separate organization. To obtain a list of private providers, the starting point was a database of facilities maintained by the UP Nursing Home Association, which was supplemented by interviews with local doctors, medical colleges, and professional associations, and then confirmed by conducting a census of providers in the selected districts to confirm their presence. In each of the three districts, four large private hospitals, 22 small private hospitals, and 28 solo practitioners were randomly selected, with doctors and nurses selected at each facility from complete lists of doctors and nurses using the same approach as in AP. Experienced data collectors were trained in the study protocols, along with standardized reference manuals and supervision. Nearly all the data were collected in one or two visits to a facility, but up to six visits were conducted to ensure data completeness. Further details of the full study methods are reported elsewhere [36,37].

A 17-item instrument was developed based on the JDI [32] to assess the importance (in an ideal job) and actual presence (in current job) of attributes specific to the job and their work environment as a measure of job satisfaction [31]. The questionnaires were pilot tested in five health care institutions of different types and used to re-phrase the items. Fifteen items cover five main areas, namely: (a) work, (b) supervision, (c) pay, (d) promotions, and (e) coworkers. Because of the concerns about governance and corruption in the health sector, and the expectation that these concerns may differ between AP and UP, and across public and private sectors [12], items were added to examine these aspects of the work environment: one item on political interference and the other on bribery. Each of the 17 items was presented to the selected doctors and nurses for self-administration. Health workers were first asked to give their opinion as to how important the 17 factors are for the ideal job. The five point rating scale consisted of: (0) of no importance; (1) of little importance; (2) of some importance; (3) very important; and (4) extremely important. The same items were used again, the second time asking the health workers to rate the extent to which these factors were present in their job. The five point rating scale consisted of: (0) not there at all; (1) present a little; (2) present to a small extent; (3) present to a large extent; and (4) fully present. We conducted exploratory analysis analyzing the Likert scales as continuous, ordinal, and binary variables (created from the highest two outcomes - (3) & (4) - as "Important"/"Present" compared with the low and neutral responses - (0) to (2) - as "Not Important"/"Not Present"). Since the results were similar, we show the binary outcome results here for simplicity. Internal consistency of the scales was assessed by Cronbach's alpha, and found to be adequate: 0.76 for the ideal scale, and 0.79 for the actual scale. To show the extent of discrepancy between perceived importance and actual presence of each item, we created a dichotomous variable for discordance for each attribute whenever an attribute was thought to be "Important" but "Not Present".

Descriptive analysis was performed to delineate the gender, age, job (doctor or nurse) and public/private affiliation of the sample. We conducted a principal components analysis on the importance ratings (in an ideal job) to examine the structure of the responses and determine whether unique patterns of items can be identified to determine whether the 17 "ideal job" attributes could be collapsed into common categories of health worker preferences. The principal components analysis involved principal component extraction with a Varimax rotation done initially on each of the four groups (public/private in AP, UP) and then the combined sample.

Logistic regression models were used for each binary outcome variable to test the significance of differences in the public vs. private sectors for each state adjusting for sex, job, and age category using dummy indicator variables in the following equation:

$$\text{Log }\left[{\text{P}}_{\text{job characteristic present}}/\left(\text{1}-{\text{P}}_{\text{job characteristic present}}\right)\right]={\text{B}}_{0}+{\text{ B}}_{\text{1}}\left(\text{Public}/\text{Private}\right)+{\text{B}}_{\text{2}}\text{Sex}+{\text{B}}_{\text{3}}\text{Job}+{\text{B}}_{\text{4}}\text{Age category}$$

Wilcoxon signed rank tests were used to make comparisons between the ratings of public and private sector workers within each state.

## Results

### Demographics of health workers

A total of 1916 health workers completed the questionnaire: 621 from AP and 1295 from UP. Although four private facilities refused to participate in the study (1.2%), we were able to obtain 100% response rate from the health care workers at participating facilities. Missing data for individual items ranged from 0.3% to 3.5%, which were dropped from the analysis. Forty-seven per cent of respondents in AP were employed in the public sector, while 68% of respondents in UP were public sector health workers. In both states, and within each employment group (public sector and private sector), a majority of respondents were male, ranging from 52% of public sector respondents in AP to two-thirds of public sector respondents in UP. Most respondents were between 30 and 45 years of age, and most were doctors, with a range from 65.2% doctors (vs. nurses) in the UP public sector to 82.4% in the AP public sector. Further details on respondent demographics are provided in Table 1 below.

**Table 1:** Characteristics of health providers

	STATE (AP)		STATE (UP)	
	
	Public	Private	Public	Private
	n = 290	n = 331	n = 875	n = 420
**Sex**				
Male	55.2%	57.7%	66.7%	58.6%
Female	44.8%	42.3%	33.3%	41.4%

**Age**				
< 30 Years	10.7%	23.9%	5.6%	20.7%
30-45 Years	68.6%	50.5%	69.0%	58.3%
> 45 Years	20.7%	25.7%	25.4%	21.0%

**Position**				
Doctor	82.4%	74.0%	65.2%	79.1%
Nurse	17.6%	26.0%	34.8%	21.0%

### Identification of main factors of job characteristics

The principal component analysis yielded four distinct components with Eigenvalues greater than one for the combined sample and for the UP-Public sector sample, whereas the UP-Private sector, AP-Public sector, and AP-Private sector samples had five components. Yet the patterns of items for components were very similar across groups when the analysis of each group was limited to four components, so the combined sample is used to describe the components (Table 2). The first component contains factors that relate to the nature of the job itself and the work environment, including attributes related to the challenging nature of the work, physical conditions of work, relationships with colleagues, and preparedness for the task at hand. The second component relates more to the extrinsic benefits of the job, including income, employment benefits, time for family life, and job location. The third category emerging from the principal component analysis includes autonomy and job security. Finally, the last category relates to transparency, and includes not having to pay bribes to get what is desired and not having one's work influenced by political decisions. We therefore refer to the four ideal job preference categories as 'job content & work environment', 'extrinsic benefits,' 'autonomy & job security,' and 'transparency'.

**Table 2:** Principal component analysis of importance ratings of job characteristics by health providers in both states (public and private)

Rotated component matrix				
	**Component correlation**			
	1 Job content & work environment	2 Extrinsic benefits	3 Autonomy & security	4 Transparency
Challenging work	**0.714**	-0.028	0.095	0.039
Training opportunities	**0.680**	0.158	-0.064	-0.007
Tools to use skills on the job	**0.667**	0.202	0.131	0.137
Good working relationship with colleagues	**0.602**	0.177	-0.025	-0.053
Good physical conditions	**0.591**	0.203	0.028	0.072
Knowing what you are expected to do	**0.511**	0.280	0.156	0.079
Good employment benefits	0.171	**0.748**	0.128	-0.040
Good income	0.013	**0.644**	0.048	0.142
Opportunity to advance	0.360	**0.617**	0.125	-0.043
Time for family life	0.089	**0.563**	-0.093	0.039
Based in good location	0.343	**0.520**	0.082	-0.082
Keeping job as long as you want	0.081	0.148	**0.735**	0.078
Independence from interference	0.046	0.017	**0.727**	-0.174
Not having work influenced by political decisions	0.002	0.038	-0.270	**0.666**
Not having to pay bribes to get what you want	0.099	-0.020	0.107	**0.787**
Superior who recognized work	0.351	0.488	0.228	-0.015
Trusted by clients	0.460	0.147	0.335	0.378

Notes: Extraction method: principal component analysis.

Rotation method: varimax with Kaiser normalization.

Attributes in bold indicate correlation of 0.5 or greater

### Importance ratings for ideal job characteristics

Nearly all items on the ideal job attributes instrument received high average scores, with the per cent rated as "important" (3 or 4 on the 0 to 4 scale) greater than 75% for all items other than "keeping job as long as you want" and "independence form interference"(Table 3). The five job characteristics with the highest importance rating overall were "good working relationships with colleagues", "physical conditions", "training opportunities", "tools to use skills on the job", and "challenging work", all of which are included in the 'job content and work environment' component. Contrary to what might be expected according to popular belief, "good income" was not rated in the top ten most important job characteristics overall; it was the third *least *important characteristic of an ideal job, according to respondents. Perceptions of ideal job characteristic were fairly similar across sectors in AP. In this state, doctors and nurses in both public and private sectors had the same top five important job characteristics ("good working relationships with colleagues" and "good physical conditions" were numbers 1 and 2, respectively, with the order of numbers 3-5 varying slightly). In UP, both public and private sector workers gave the highest ratings to "good working relationships with colleagues". However, in the UP public sector, "trusted by clients" and "opportunity to advance" also made it into the top five most important job characteristics (along with "tools to use skills on the job and "training opportunities"). In the UP private sector, "trusted by clients" was also in the top five items of importance, along with "good physical conditions", "training opportunities," and "tools to use skills on the job".

**Table 3:** Percent of health providers rating job characteristics as important, by state and public/private sector

INDICATOR	STATE(AP)		STATE(UP)	
	
	Public	Private	Public	Private
**Job content & work environment**				
Challenging work	88.6	83.9	**93.3**	91.6*
Training opportunities	**90.3**	81.5**	96.0	93.2
Tools to use skills on the job	83.4	84.2	**96.8**	93**
Good working relationships with colleagues	93.8	90.6	98.7	97.8
Good physical conditions	90.7	90.6	93.5	94.2
Knowing what you are expected to do	**83.0**	72.3**	92.3	89.6
**Extrinsic benefits**				
Good employment benefits	**76.2**	53.3***	**94.0**	65.8***
Good income	74.4	66.5	**82.1**	73.4***
Opportunity to advance	72.0	65.3	**94.3**	77.7***
Time for family life	77.9	75.8	**93.9**	85.5***
Based in good location	**83.1**	72.5**	**93.8**	84.3***
**Autonomy & security**				
Keeping job as long as you want	57.2	52.7	70.2	73.3
Independence from interference	58.9	49.7*	56.0	61.6
**Transparency**				
Not having to pay bribes to get what you want	75.5	74.1	85.5	87.9
Not having work influenced by political decisions	80.3	79.5	**74.0**	85.9***
**Other**				
Trusted by clients	70.6	74.4	94.8	93.2
Superior who recognizes work	**74.7**	60.7***	**92.9**	68.8***

* *P *< 0.05

** *P *< 0.01

*** *P *< 0.001

Note: results adjusted for sex, age, and profession

The most important items for public and private sector workers in both states involved 'job content and work environment' factors. However, there were also differences between public and private sector workers. In both states, the largest difference between public and private sector workers involved factors related to extrinsic benefits. "Good employment benefits", was rated much higher in importance in the public sector than the private sector (76% compared to 53% in AP, *P *< 0.001; 94% compared to 66% in UP, *P *< 0.001). Highly significant differences between public and private sectors across both states were evident for "superior who recognizes work" and "based in a good location". In AP, public sector workers also rated "knowing what you are expected to do" (83% compared to 72%, p < 0.01), "training opportunities" (90% compared to 82%, p < 0.001) and "independence from interference" (59% compared to 50%, p < 0.01) significantly higher than their private sector counterparts. In UP, public sector workers rated as significantly higher than the private sector "time for family life", "tools to use skills on the job," "challenging work," "good income", and "opportunity to advance." Transparency factors were also more important to private sector workers than public sector workers in UP, as they rated "not having work influenced by political decisions" as significantly more important (See Table 3).

### Ratings of actual presence of characteristics in current job

Analysis of the perceived actual presence of the same 17 job characteristics also revealed differences by state and by public and private sector workers (Table 4). Actual presence of most job attributes was lower than the perceived importance, across both states and sectors. In AP, actual conditions were rated as better in the private sector than the public sector for 13 of the 17 attributes (Wilcoxon signed-rank test p < 0.05), whereas in UP, they were rated as better in the private sector for 15 of the 17 attributes (Wilcoxon signed rank test p < 0.01). In both UP and AP, private sector workers rated "training opportunities" as more present than public sector workers (though this difference was only significant in AP), along with "tools to use skills on the job", "not having work influenced by political decisions", "trusted by clients", and "based in good location". Private sector workers in the UP rated nearly all items as more present than their public sector counterparts. The only item where there was no difference between public and private sector in UP involved "good income", which was also not different in AP. The only areas where public sector workers rated present conditions as significantly better than in the private sector involved "good employment benefits" and "superior who recognized work", which were higher in the public sector in both states.

**Table 4:** Percentage of health providers rating job characteristics as present, by state and public/private

INDICATOR	STATE(AP)		STATE(UP)	
	
	Public	Private	Public	Private
**Job content & work environment**				
Challenging work	61.4	62.3	**54.0**	74.0***
Training opportunities	41.3	52.3	**29.3**	49.0***
Tools to use skills on the job	**34.8**	55.3***	**37.6**	75.5***
Good working relationships with colleagues	83.4	79.0	**80.5**	90.1***
Good physical conditions	54.2	61.5	**43.6**	78.7***
Knowing what you are expected to do	61.2	62.1	**63.4**	79.5***
**Extrinsic benefits**				
Good employment benefits	**43.1**	22.5***	**45.3**	22.2***
Good income	34.6	39.7	43.5	45.2
Opportunity to advance	33.7	36.5	**34.1**	41.4*
Time for family life	32.9	38.5	**47.4**	55.0*
Based in good location	**47.8**	56.9*	**35.2**	78.7***
**Autonomy & security**				
Keeping job as long as you want	45.5	50.5	**49.3**	71.7***
Independence from interference	37.4	41.8	**39.6**	49.9***
**Transparency**				
Not having to pay bribes to get what you want	72.8	72.7	**70.0**	89.1***
Not having work influenced by political decisions	**70.2**	79.8**	**42.8**	89.0***
**Other**				
Trusted by clients	**56.7**	69.3**	**78.5**	91.5***
Superior who recognizes work	**50.3**	43.6*	**53.0**	43.4*

* *P *< 0.05

** *P *< 0.01

*** *P *< 0.001

Note: results adjusted for sex, age, and profession

### Discordance between importance and actual presence of job characteristics

A summary of the discordance scores is shown in Table 5. The good news is that the most highly ranked item of importance - "good working relationships with colleagues" - was usually present (missing for only 16% of health workers), indicating that health workers for the most part are experiencing good relationships with colleagues they highly value. However, there are numerous findings of concern. One is the relatively high levels of discordance, with the highest discordance rates occurring in three of the items considered among the most important: "training opportunities" (55% discordance overall), "opportunity to advance" (50% overall), and "tools to use skills on the job" (47% overall). Public sector workers also had higher levels of discordance than private sector workers. The public sector discordance scores were significantly higher for all indicators in UP (Wilcoxon signed rank test *P *= 0.0003), and all but one indicator in AP (Wilcoxon signed rank test *P *= 0.0004). There were particularly large differences (greater than 30 percentage points) between public and private sectors in the UP, including "based in a good location", "good physical conditions", and "not having work influenced by political decisions" (see Table 5).

**Table 5:** Percentage of health providers rating job characteristics as discordant, by state and public/private (discordant = important but not present) †

INDICATOR	STATE (AP)		STATE (UP)	
	
	Public	Private	Public	Private
**Job content & work environment**				
**Challenging work**	**31.8%**	**26.3%**	**41.1%**	20.0%***
**Training opportunities**	**51.0%**	33.4%***	**67.7%**	46.9%***
**Tools to use skills on the job**	**54.3%**	35.4%***	**59.8%**	22.2%***
**Good working relationships with colleagues**	**13.8%**	**16.8%**	**19.0%**	9.2%***
**Good physical conditions**	**39.2%**	**31.2%**	**51.2%**	18.0%***
Knowing what you are expected to do	29.1%	16.4%***	31.5%	16.7%***
**Extrinsic benefits**				
Good employment benefits	39.2%	34.6%	50.0%	47.2%**
Good income	44.8%	33.6%*	44.8%	36.3%***
Opportunity to advance	43.9%	34.0%**	61.4%	42.2%***
Time for family life	47.8%	42.6%	49.2%	35.3%***
Based in good location	39.8%	21.9%**	60.2%	13.9%***
**Autonomy & security**				
Keeping job as long as you want	23.4%	16.6%**	26.1%	15.1%***
Independence from interference	32.2%	19.2%**	28.1%	23.5%*
**Transparency**				
Not having to pay bribes to get what you want	10.5%	6.0%*	20.5%	4.7%***
Not having work influenced by political decisions	16.6%	8.0%**	37.5%	5.2%***
**Other**				
Trusted by clients	18.4%	11.0%**	18.4%	7.1%***
Superior who recognizes work	34.6%	24.8%**	41.4%	31.6%**

† In bold: attributes with the top 5 importance ratings

* *P *< 0.05

** *P *< 0.01

*** *P *< 0.001

Note: results adjusted for sex, age, and profession

## Discussion

### Main factors describing health worker satisfaction

The factor analysis demonstrated that groupings of variables are largely consistent with earlier results on worker satisfaction, but did not correspond exactly to the indices on which the instrument was based. Our results show that while work and co-worker factors have merged into one group, pay and promotions have merged into another group. The three items under supervision were distributed into different groups. One supervision item, namely "knowing what you are expected to do" merged with the job content and work environment group, the other item "superior who recognized work" remained separate. Its correlation with the second component of extrinsic benefits (0.488) was slightly higher than its correlation with the first component (0.351) of 'job content and work environment'. Less interference by superiors, the third item under supervision emerged in a distinct group along with job security. The sixth group of items on Transparency remained separate. These findings suggest that investigations on worker satisfaction and motivation should be customized to the group under investigation, and that assumptions about motivating factors should not be based exclusively on existing theories.

Contrary to common perceptions, many more employees rated motivating factors like "good working relationships with colleagues" (96%), "training opportunities" (92%), and environmental factors, such as having "tools to use skills" (92%), and "good physical conditions" (93%) as more important than income (76%). It is possible that respondents did not want to appear selfish and report remuneration as a higher motivating factor, even on a self-reporting instrument. However, in Ghana, health workers overwhelmingly identified low salaries as the main source of dissatisfaction on an interviewer-administered questionnaire [38]. In AP and UP, it may also be that those workers for whom remuneration is most important may be more likely to be absent from work in order to pursue other sources of income, particularly in the public sector. Alternatively, public health worker incomes may be comparable to what they can earn in the private labour market, and their expectations are influenced by this. Nonetheless, the prominence of non-financial motivating factors make it difficult to argue that better salaries alone will significantly improve health worker motivation, as other studies in Africa have begun to conclude [39,40].

Our findings on the importance ratings of various job characteristics are also largely consistent with findings from other studies conducted in the United States. For example, Cashman and colleagues found that doctors wanted autonomy and job status above a high income, and regarded extrinsic benefits as less important [41]. Gray concluded that extrinsic benefits did not feature prominently among nurses, while improved communications with and a caring boss were considered important [42]. But these nurses wanted better monetary compensation more than anything else. Another study of nurses by Tumulty, Jernigan, and Kohut found intrinsic motivating factors to be more important than extrinsic ones [43]. Yet another study on the nursing profession found that the major motivating factors for nurses were recognition, the work itself and responsibility [44].

A review of 12 empirical studies of motivation in both developing and developed countries found that seven major job characteristics were important determinants of motivation (work itself; relationships at work; workplace conditions; opportunities for personal development; pay/rewards; management practices; and organizational policies), but the relative importance of these factors varied widely depending on the setting and methodologies used [22]. A study in Vietnam, (included in the review just cited) found that the main motivating factors for health workers were appreciation by managers, colleagues and the community, a stable job, income, and training, while the primary factors for dissatisfaction were low salaries and difficult working conditions [45]. Another study in Jordan and Georgia, also included in the review by Dolea and Adams, found that the two countries exhibited many similarities among key motivational determinants, including self-efficacy, pride, management openness, job properties, and values; however, some divergent results indicated the importance of local culture on motivational issues [46]. This interpretation is consistent with the findings in AP and UP.

### Differences in employment environment between the two states

Employee ratings of the importance and actual presence of various job characteristics is an indicator of differences in the human resource environment of the respondents. The analysis of health worker ratings of importance and presence of specific job characteristics in the UP and AP health sectors showed that context, specifically geopolitical setting and employment sector (public versus private), is extremely important in assessing health worker satisfaction. Overall, importance ratings tended to be higher in UP compared to AP, particularly for employment benefits and good income, opportunity to advance, time for family life, being based in a good location, keeping the job as long as you want, not having to pay bribes to get what you want, trust by clients, and having a superior who recognizes one's work.

### Differences in employment environment between private and public sector

Differences in employee ratings of the importance and actual presence of various job characteristics also give us some idea about the difference of employment environments in the private and public sectors. Public sector and private sector contexts are different, both in terms of the importance workers place on various job characteristics, as well as the actual presence of these factors. This is likely because there are differences in the characteristics of health providers who choose to work in the private sector versus the public sector, as well as differences in the actual working conditions - the organizational factors and culture - in the public and private sector within each state. Individuals who choose to work in the public versus the private sector may have different personal values, and therefore choose organizational/workplace cultures that are compatible with these values. For example, there is much greater freedom to decide the location of work in the private sector compared to the public sector, and within the public sector, it is our impression that health workers had a better chance of being posted in a location of their choice in AP rather than UP. Our analysis also shows that "not having work influenced by political decisions" was rated more important for private sector UP workers than public sector workers (86% compared to 74%, *P *< 0.001). This factor was also much more present in the private compared to public sector (89% compared to 43%), indicating compatibility (little discordance) between personal value and organizational culture.

The fact that "not having work influenced by political decisions" was rated as significantly more important by private sector in comparison to public sector workers in UP may reflect the high degree of political influence and lack of transparency in the public sector. Health workers may choose to operate in the private sector in UP because they value freedom from government interference and corruption, and trust from clients, more than their public sector counterparts. In general, self-reported job conditions were better overall in the private sector than in the public sector, particularly in the UP, where major differences emerged. In the UP, the private sector rated all indicators but three as more present than their public sector counterparts. The biggest advantages, greater than 20 percentage points, that UP private sector workers reported over public sector workers related to the presence of: challenging work, good physical conditions, tools to use skills on the job, keeping job as long as you want, not having work influenced by political decisions, and being based in a good location. However, public sector workers perceived significantly better employment benefits (both UP and AP) and increased presence of a superior who recognized their work (UP). On most indicators, AP public sector health workers rated conditions better than their public sector colleagues in UP. However, private sector workers in UP rated conditions more favourably on most indicators than AP private sector workers.

### Which health workers have the biggest gap in expectations?

Discordance scores tell us which indicators have the biggest gap in perceived importance and actual presence, and therefore should be useful for indicating which strategies to improve job satisfaction may have the biggest impact. These strategies should not only target job factors felt to be important by workers, but also important job conditions with high discordance scores. Since discordance was particularly striking in the public sector, it is much more important for public sector managers to pay attention to these issues in these two states. One strategy may be to find ways to learn from the private sector, and perhaps replicate some of the relevant conditions found in the market, such as by giving more autonomy to public health workers over questions of location or work, or by providing transparent incentives to work in difficult locations.

The four most important indicators are factors that are somewhat amenable to change. For example, increasing training opportunities, improving the physical working conditions and environment through improved physical structures, equipment, and materials, may help improve these important working conditions. Other highly discordant factors (greater than 40% discordance) included opportunity to advance, good employment benefits, time for family life, good income, and being based in a good location. These are also issues where policymakers and managers can intervene to close the gap between importance and presence.

### What are the limitations of this study?

This study was designed as an exploratory examination of health worker satisfaction in two Indian states. As such, it provides a snapshot of health worker perspectives at one point in time, and the causal relationships between work conditions, satisfaction, and motivation cannot be further delineated in this study. This study did not measure performance of health workers, so it is not clear how the reported factors relate to their actual performance on the job or to job tenure. Such analyses will be important to managers and policy-makers, who may see job performance as the most important outcome. Researchers have also posited that health worker motivation is clearly linked to workers' performance as well as intention to quit [22]. The type of motivational variables analyzed in this paper here may well be important determinants of job retention and performance, though future studies should be conducted to assess whether this proves to be the case. Finally, the analyses done in this study were relatively crude, limited in part by datasets that did not allow for clustering of health workers at facilities, having a limited number of variables to examine, and by compressing responses into bivariate categories which may lose some information.

## Conclusions

The findings of this paper reflect conditions for public and privately employed health workers in two states in India, and are therefore most relevant to health workers there. The findings point towards the following when it comes to improving job characteristics that matter to health workers.

• Improvements in training opportunities for skill development and availability of equipment for effective use of existing professional skills have the greatest promise to raise health worker satisfaction in both states. The public sector in particular needs to focus attention on this aspect of their health sector human resource management, since the level of discordance on this aspect was significantly more in the public sector compared to the private sector.

• Whereas a large number (41%) of public sector health workers in both states had discordant views about their work location, the problem appears to be particularly acute in UP. Some useful interventions in this area may be: (a) reorganization of health worker cadres into smaller geographical entities, so that potential candidates can self select geographic cadres according to their personal preferences, (b) transparent, responsive and reliable transfer and posting policy.

• Even though the financial package was rated as lower in importance compared to non-financial incentives related to job content and work environment, employers are well advised to not ignore the incomes of health workers. Overall between 40 to 50% of health workers did not realize their salary expectations.

• The organizational culture requires attention. Training and motivation of supervisory personnel to promptly recognize good work, and foster an environment that encourages autonomy will improve health worker satisfaction and motivation. Here again the need appears greater for the public sector institutions. About 41% of public sector health workers in UP reported discordance due to inadequate recognition of their work. In AP about 35% of public sector health workers longed for supervisors who would recognize their work.

• For health workers engaged in the public sector, the effect of corruption on their day to day working may need closer analysis. It provides further incentive to policy-makers and managers to take steps to reduce corruption, though this may not be always easy. Trying to reduce the effect of corruption on the morale of workers may be an approach to follow. This would entail involving workers in identifying problems with non-transparency and its impact on them, and working with them to reduce its effects.

Our study of different types of health workers in two states suggests that understanding motivation and satisfaction of health workers is highly dependent on the local context. Perhaps one of the most important implications of this is that health managers ought to be asking their own workers about their particular motivational factors, and developing plans locally to address them.

## Competing interests

The authors declare that they have no competing interests.

## Authors' contributions

DHP conceived of the study, participated in the design and coordination, and helped to draft the manuscript. SC and MP participated in the conceptualization and design of the study and helped to draft the manuscript. SC led the study team in Uttar Pradesh, overseeing the initial analysis of UP state data, while MP did the same for the team in Andhra Pradesh. LS undertook the combined analysis, and contributed to the drafting of the manuscript. All authors read and approved the final manuscript.
